# An Optimized Mechanical Peeling Protocol for Mouse Corneal Endothelium

**DOI:** 10.3390/bioengineering13080869

**Published:** 2026-07-28

**Authors:** Zhenwei Song, Liujiang Song, Hua Mei

**Affiliations:** 1 Division of Pharmacotherapy and Experimental Therapeutics, Eshelman School of Pharmacy, The University of North Carolina at Chapel Hill, 301 Pharmacy Ln, Chapel Hill, NC 27599, USA; zhenwei_song@med.unc.edu; 2 Center for Molecular Medicine, The University of North Carolina at Chapel Hill, 104 Manning Dr, Chapel Hill, NC 27599, USA; 3 Department of Ophthalmology, The University of North Carolina at Chapel Hill, 115 Mason Farm Rd, Chapel Hill, NC 27599, USA; 4 Biological Chemistry & Pharmacology, The Ohio State University, 2255 Kenny Road, Columbus, OH 43210, USA; liujiang.song@osumc.edu

**Keywords:** mouse, corneal endothelium, corneal endothelial cells, whole mount, mechanical peeling, isolation method

## Abstract

Corneal endothelium is a single layer of polygonal cells responsible for maintaining stromal dehydration and corneal transparency. Isolating corneal endothelium from murine models is exceptionally challenging without an established protocol, which historically limits the utility of mouse models to study corneal endothelial disease. Herein, a detailed method is presented to obtain a highly pure population of corneal endothelial cells via mechanical peeling of the endothelial sheet from both young and aged mice. Under a dissecting microscope, the corneal dome, including the limbus, was dissected. Following the removal of the iris and trabecular meshwork, the corneal dome was rinsed in phosphate-buffered saline (PBS). Fine, blunt forceps were utilized to stabilize the edge of the corneal dome, while ultra-fine forceps were employed to grasp the edge of Descemet’s membrane (DM). The edge of the DM was identified by performing a gentle micro-scratch along the limbal margin until a small flap emerged. Subsequently, the entire endothelial sheet along with the DM was carefully peeled from the periphery toward the corneal center. The isolated endothelial sheet can be dried flat on a microscope slide to investigate spatial gene expression, cell morphology, and cellular interactions. To obtain a single-cell suspension, the endothelial sheet was enzymatically digested in 0.25% Trypsin-EDTA for 8 min at 37 °C, yielding a highly purified population of corneal endothelial cells (95.7%). This protocol demonstrated high reproducibility for both young (approximately 1 month old) and aged (approximately 1 year old) mouse corneas. The total cell yields collected from young and old mice were 3634 and 4560 cells per eye, respectively, exhibiting high cell viability (young: 98.3%, old: 95.8%) and a high percentage of single cells (young: 90.6%, old: 90.1%). This detailed isolation protocol will facilitate advanced downstream research on the corneal endothelium using murine models.

## 1. Introduction

The corneal endothelium is a monolayer of geometrically polygonal cells adhering to the Descemet’s membrane (DM). This specialized cell layer is essential for maintaining corneal transparency and appropriate stromal dehydration [1], but it lacks typical regenerative capacity in vivo during aging [2], upon wounding [3,4], or following pathologic damage [5,6,7]. The progressive aged or acute loss of healthy corneal endothelial cells leads to severe stromal overhydration, a swollen cornea, and a partial or total loss of vision. Endothelial layer dysfunction represents a primary manifestation in a major percentage of corneal diseases [8,9]. These encompass Fuchs’ endothelial corneal dystrophy (FECD), posterior polymorphous corneal dystrophy (PPCD), aphakic or pseudophakic bullous keratopathy (ABK/PBK), endothelial dysfunction caused by penetrating or blunt trauma, congenital hereditary endothelial dystrophy (CHED), iridocorneal endothelial (ICE) syndrome, previous failed corneal grafts, and herpes simplex virus endotheliitis.

At present, the clinical management of dysfunctional endothelium heavily relies on the transplantation of healthy human donor tissue [10]. However, global donor tissue shortages have driven tissue bioengineering through cultivation endothelium provide an alternative cell therapy strategy for this replacement [11]. Various in vitro expansion methods for corneal endothelial culture have been established, significantly expanding both laboratory research capacity and clinical application pipelines [12]. Consequently, a majority of investigations into corneal endothelial cells and related disease pathophysiologies are conducted using in vitro cultured corneal endothelial cells across multiple species (including rabbit [13,14], mouse [15,16,17], human [18,19], monkey [20], bovine [21,22]) or via in vivo animal models [23,24,25,26,27,28,29,30,31]. The growing pre-clinical testing for novel endothelial therapies calls for highly predictive in vivo frameworks [24,32], and small animal models have become well established [33]. Among these, murine models are exceptionally popular due to their lower husbandry costs, rapid breeding cycles, and the widespread availability of advanced analytical tools, including transgenic mouse lines. This highly homogeneous genetic background makes mouse models uniquely attractive, rendering them the preferred choice for exploring complex genetic diseases like corneal endothelial dystrophy (CED) or FECD.

Investigating latent proliferation and identifying potential endothelial progenitor populations are highly promising approaches for developing advanced bioengineered corneal endothelium [34]. Recently, high-resolution single-cell RNA sequencing (scRNA-seq) has revealed significant, previously unrecognized cellular heterogeneity within the corneal endothelial layer [35]. Furthermore, evaluating full flat mounts of this intact monolayer can provide crucial insights into spatial gene expression, active cell migration dynamics, and age-related distribution changes [36] across the corneal endothelium anatomy. To fulfill these analytical advances, a single and highly purified cell population serves as the crucial foundation for facilitating high-resolution downstream investigations. However, achieving this degree of purification remains exceptionally challenging. While numerous research groups have utilized mouse models to study corneal endothelium regeneration [18], isolating the tissue as a critical starting material, most investigators harvest the murine corneal endothelium by simply stripping the DM–endothelial complex under a dissection microscope [16,37,38,39]. However, a detailed, standardized protocol optimized for mechanical corneal endothelial collection from mouse eyes remains unavailable in the literature.

Here, to address this technical gap, a detailed micro-surgical protocol is introduced to isolate intact corneal endothelial sheets suitable for high-fidelity whole-mount and single-cell analysis from both young (approximately 1 month old) and aged (approximately 1 year old) murine eyes. Crucial baseline parameters, including final cell yield, viability, phenotypic purity, and the percentage of single cells collected per mouse eye were systemically examined. This standardized isolation protocol is intended to provide a reliable technical baseline to facilitate advanced downstream molecular and translational research of the corneal endothelium using murine models.

## 2. Material and Methods

**Figure 1:**
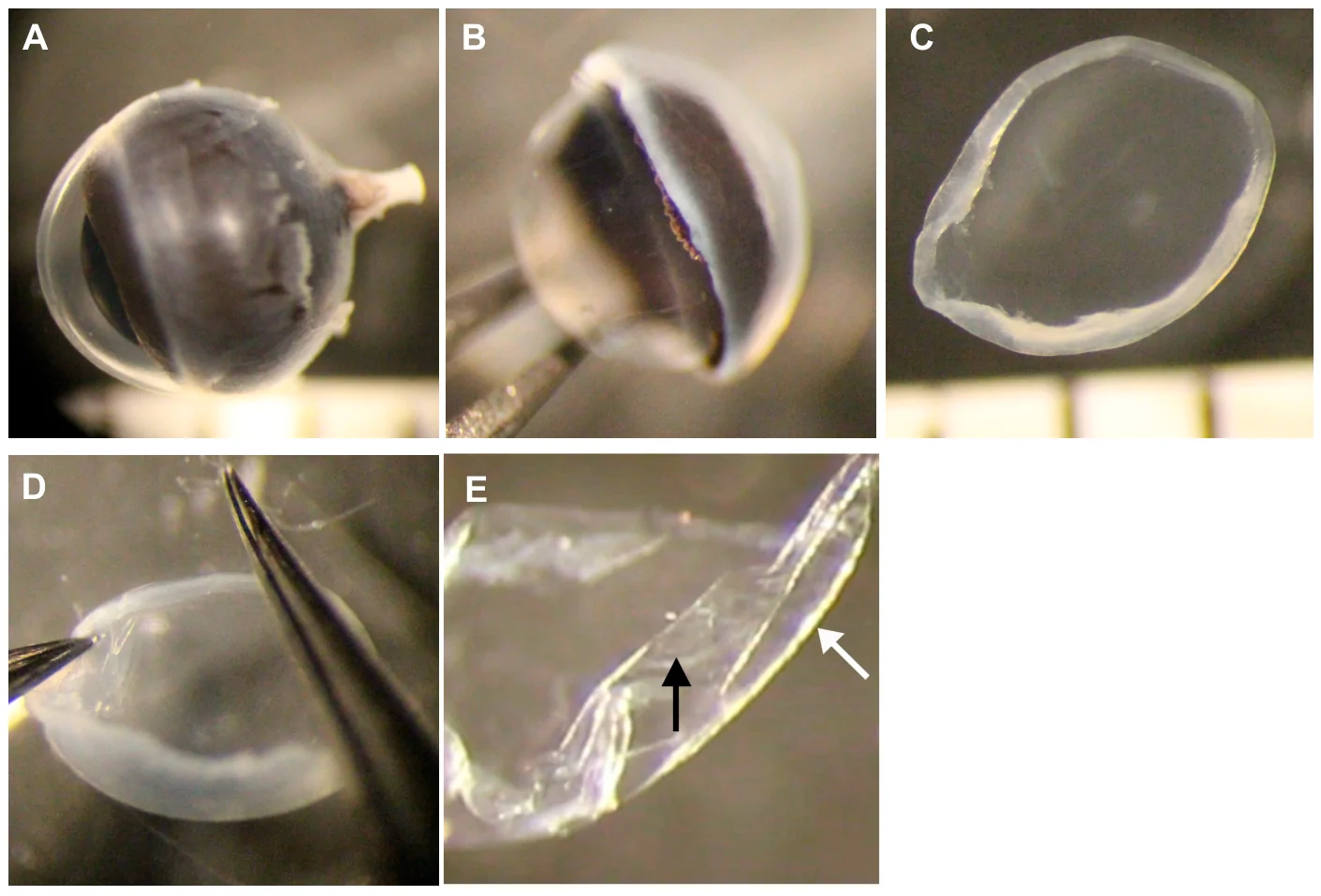
Micro-surgical isolation and physical orientation for mouse endothelial mechanical peeling. (**A**) Initial ocular globe maintenance in ice-cold complete medium following enucleation. (**B**) Stereomicroscopic image displaying the stabilization of the posterior eye globe using fine forceps and the circumferential incision along the limbal rim to isolate the intact corneal dome. (**C**) Internal surface cleaning of the isolated corneal dome following iris removal. (**D**) Visualization of the flapped initiation site during the mechanical peeling of the endothelium. One pair of forceps is utilized to fix the corneal dome in place, while a second pair is employed to detach the peripheral endothelium. (**E**) Determination of tissue orientation utilizing the peeled endothelium–Descemet’s membrane (DM) complex floating in PBS. Against light reflection during gentle shaking, the endothelial cell surface facing upward appears less transparent, rougher, and exhibits a dimmer light reflection (black arrow). Conversely, the acellular DM surface appears significantly smoother and displays a brighter, highly specular reflection (white arrow).

1. Animal: Wild-type mice (C57BL/6J; Jackson Laboratory, Bar Harbor, ME, USA; Cat# 000664) aged 1 month (young) and 12 months (aged), as well as a TetOP-H2B-GFP mouse (Jackson Laboratory, Bar Harbor, ME, USA; Cat# 016836) at 5–6 weeks of age were utilized in this study. All animal procedures and experimental protocols were reviewed and formally approved by the Institutional Animal Care and Use Committee (IACUC) at the University of North Carolina at Chapel Hill under protocol number #18-024. All housing, environment, and husbandry conditions complied strictly with the Association for Research in Vision and Ophthalmology (ARVO) Statement for the Use of Animals in Ophthalmic and Vision Research.
2. Cell/Organ Culture Medium: The culture medium was composed of DMEM/F12 medium (Gibco, Grand Island, NE, USA;Cat# 11330032) supplemented with 20% fetal bovine serum (FBS, Sigma, St. Louis, MO, USA; Cat# TMS-013-B), 100 U/mL penicillin and 100 μg/mL streptomycin (Gibco, Grand Island, NE, USA; Cat# 15-140-122), 10 μg/mL gentamicin, and 0.25 μg/mL amphotericin B (Fisher, Waltham, MA, USA; Cat# NC0308445).
3. Equipments: All micro-isolation procedures were executed under a sterile stereomicroscope environment using a Leica MZ6 stereomicroscope (Leica Microsystems, Wetzlar, Germany) equipped with a Canon EOS Rebel T5i digital camera (Canon Inc., Tokyo, Japan) for high-resolution digital documentation. All specialized micro-surgical instruments, forceps, and complementary materials are compiled with manufacturing details in Supplementary Table S1.
4. Corneal dome preparation and cleaning: Mice were humanely euthanized in accordance with approved IACUC guidelines. Enucleation of the ocular globe was achieved by utilizing 15° curved micro-forceps (Sklar Surgical Instruments, West Chester, PA, USA; Cat# 82030-028, 19-1045) to gently apply downward pressure on the nasal aspect of the eyelid, facilitating the excision of the intact eyeball along with minimal adjacent adnexal tissue. The enucleated globe was immediately transferred into a 35 mm sterile Petri dish (Corning Inc., Corning, NY, USA; Cat# 353001) filled with ice-cold, sterile phosphate-buffered saline (PBS; Sigma-Aldrich, St. Louis, MO, USA; Cat# D8662). Adhering eyelid tissue and extrinsic ocular muscles were meticulously excised using curved spring scissors (Fine Science Tools, Foster City, CA, USA; Cat#15004-08) and fine non-toothed forceps (Dumont #5; Fine Science Tools, Foster City, CA, USA; Cat#11295-20) without making physical contact with the corneal dome. The cornea was then separated from the posterior segment by circumferentially incising along the distinct limbal rim.
5. Peeling of corneal endothelium sheet: The isolation of the mouse corneal endothelium was executed via a sequence of micro-surgical steps optimized for structural preservation and reproducibility.
  Step1: Orientation: To initiate the peeling, the first important step was to find a starting point to detach the DM. The isolated corneal dome was positioned in a sterile Petri dish with the endothelial layer facing upward. The focus of the Leica MZ6 stereomicroscope was dynamically adjusted to resolve the delicate endothelial cell monolayer at high magnification (2× to 4×).
  Step2: Limbal micro-scratching and flap initiation: to expose the Descemet’s membrane (DM), an ultra-fine Dumont #5 forceps tip was applied to gently scratch the extreme peripheral margin of the limbus. This localized mechanical disruption was maintained until a microscopic, translucent flap composed of the DM visibly detached from the stroma. Multiple sites could be used to initiate the DM flap from the peripheral edge.
  Step3: Circumferential Edge Detachment: to separate the DM from the stroma, the micro-scratching technique was repeated at multiple discrete clock positions along the circular limbal edge. If a loss of transparency or an increase in mechanical resistance was felt (indicating stromal adherence), the forceps were repositioned to an alternative limbal site to re-initiate a clean detaching separation. Gentle stepwise and slow detachment moving could avoid contamination from the posterior stroma.
  Step4: Mechanical peeling to harvest intact corneal endothelium sheet: With the peripheral rim fully detached, one fine forceps was used to stabilize the remaining corneal stroma, while a second forceps gently grasped the edge of the liberated endothelial–DM flap. The sheet was peeled continuously (stepwise) and slowly from the periphery toward the central region of the cornea until the complete, intact dome-shaped sheet was liberated.
  Step5: Quality check: once the sheet was released from stroma, the orientation of the floating endothelium–DM sheet was confirmed by exploiting angled light reflection under the stereomicroscope [40]. The endothelial side was readily identified by its dimmer, rougher light reflection during gentle shaking relative to the mirror-like, highly reflective DM side. Under the light, the endothelial side showed an obviously dimmer light reflection on the surface during shaking compared to the DM side (Figure 1). Alternatively, the endothelial side could be marked with trypan blue dot or punched holes [41,42,43] before peeling to facilitate the recognition of it, e.g., punch at 12 and 3 o’clock positions, “R” tattoo at the peripheral edge. Usually, the endothelial side kept a natural inward curve and did not curve the opposite way [44].

6. Corneal endothelium whole-mounting and flattening: An operation zone was established by placing a 200 μL droplet of sterile PBS onto a poly-L-lysine-coated glass microscope slide. The peeled endothelial sheet was transferred into the droplet using a 1 mL pipette tip that had been manually truncated and fire-polished to expand the orifice, preventing shear stress. The interior of the tip was pre-wetted with culture medium to prevent tissue adhesion. Under microscopic guidance, localized radial incisions were made at the peripheral edges of the sheet using fine spring scissors to convert the three-dimensional dome into a flat, “flower-shaped” geometry. A PBS droplet was carefully absorbed using lint-free task wipers (Kimwipes; Kimberly-Clark, Irving, TX, USA), leaving a thin fluid film to maintain position and hydration. Ultra-fine-tip forceps or a fine camel-hair painting brush were employed to gently unroll and stretch any curled peripheral margins. Tips: The brush tip or one tip of the forceps could be inserted into or under the curling edges and gently stirred up the edge to flat it. The unfolded edges could be further flattened by pressing and stretching gently at the peripheral areas. The areas where the brush or forceps tip touched tended to lose endothelial cells, and this effect should be minimized. We observed that a fine brush effectively flattened the outer edges of the endothelium, but could cause a more severe loss of endothelial cells than the fine-tip forceps. We recommend using the fine-tip forceps to flat the tissue or use the fine-tip brush with extra care.
7. Corneal endothelium dissociation into single-cell suspension: To generate single-cell suspensions, isolated endothelium–DM sheets were rinsed three times in sterile PBS and incubated in 500 μL 0.25% Trypsin/EDTA in 37 °C for 8 min in 1.7 mL tubes. The enzymatic reaction was quenched by adding 1 mL of complete culture medium containing 20% FBS, and the mixture was stabilized on ice for 1 min. To ensure the endothelial cells were released from DM, mechanical dissociation was completed by gently pipetting the mixture 20 times using a truncated 1000 μL pipette tip. Complete cellular detachment was confirmed when the remaining DM sheet appeared completely transparent under microscopic inspection: the DM was uniformly transparent once all cells were detached. The single-cell suspension was centrifuged at 200× *g* for 8 min at 4 °C. The resulting cell pellet was gently resuspended in 50 μL of ice-cold complete medium and maintained on ice prior to downstream quantitative analysis.
8. Immunostaining: Whole-mount slides were fixed by 4% paraformaldehyde (PFA) for 8 min at room temperature (RT) followed by three sequential 10 min rinses in PBS. Nonspecific binding sites were blocked by incubating samples for 1 h at RT in a blocking buffer consisting of 5% normal goat serum (Sigma, St. Louis, MO, USA; Cat# G6767) in PBS. The samples were incubated for 2 h at RT with primary antibodies: antibody Na+/K+-ATPase alpha1 antibody (C464.6, Santa Cruz Biotechnology, Santa Cruz, CA, USA; Cat# sc-21712) was diluted in block buffer at 1:100 and N-Cadherin (Rabbit mAb, Cell Signaling Technology, Danvers, MA, USA; Cat# 13116s) at dilution: 1:100. Following three 10 min washes in PBST (0.1% Triton X-100 (Sigma, St. Louis, MO, USA; Cat# 9344) in PBS), samples were incubated for 1 h at RT with secondary antibodies: goat anti-mouse IgG (H+L), highly cross-adsorbed secondary antibody Alexa Fluor 488 (Invitrogen, Carlsbad, CA, USA; Cat# A-11029) with dilution of 1:1000, as well as Goat anti-Rabbit IgG H&L Alexa Fluor 488 (Abcam, Cambridge, UK; Cat# ab150117) with dilution of 1:1000. Nuclei were counterstained using Hoechst 33324. And Phalloidin-Alexa Fluor 555 (Cell Signaling Technology, Danvers, MA, USA; Cat# 8953s) at 1:50 dilution was used to indicate the endothelial cells. Samples were coverslipped and imaged using a laser microscope.
9. Flow cytometry: Cell suspensions were kept continuously on ice until acquisition. A drop of propidium iodide (PI) was introduced immediately before analysis to identify and exclude dead cells. Quantitative flow cytometry was executed on a Becton Dickinson LSR Fortessa cell analyzer. Single-cell populations (singlets) were strictly gated using forward scatter area versus forward scatter height (FSC-A vs. FSC-H) and side scatter area versus side scatter height (SSC-A vs. SSC-H) parameters. Unstained cells and single-stain controls were utilized to establish baseline photomultiplier tube (PMT) voltages and compensation matrices.
10. Statistical analysis: All quantitative experimental evaluations were conducted using a minimum of *n* = 3 independent biological replicates per experimental cohort. Quantitative data are expressed as the mean ± standard error of the mean (SEM). Statistical charts, graphical figures, and associated hypothesis testings were generated using GraphPad Prism 7. Comparisons between two experimental groups were analyzed using a two-tailed Student’s t-test, whereas paired data sets were analyzed by paired *t* test. The value of *p* < 0.05 was considered as the threshold for statistically significant differences.

## 3. Results

### 3.1. Preparation of Corneal Dome for Endothelium Isolation

Although whole-mount preparation of the corneal endothelium facilitates the study of spatial gene expression, cell morphology, cell–cell interactions, and cell–matrix interactions, high-resolution downstream applications such as transcriptomic profiling and single-cell RNA sequencing (scRNA-seq) require a highly reliable method for separating the corneal endothelium.

Herein, a detailed protocol is presented to mechanically peel the endothelial cell sheet together with Descemet’s membrane (DM) and flatten it onto a microscope slide. The procedure was evaluated utilizing cohorts of both young (4 weeks old, n = 5) and aged (1 year old, n = 4) mice. Micro-dissection was executed under low-power magnification (2–4×) with tissues maintained in ice-cold PBS or complete medium to preserve cellular viability (Figure 1A).

The primary physical obstacle during isolation involved stable immobilization and immobilizing the spherical ocular globe to permit precise trimming of the corneal dome away from the eye cup. This was addressed by applying gentle traction to the optic nerve to create a small access posterior scleral perforation. By inserting one tip of a fine Dumont forceps into this perforation, the posterior pole of the globe was securely anchored. A clean incision was then extended from the posterior perforation to the limbic line using fine spring scissors, allowing the complete corneal dome to be excised cleanly along the circumferential limbal border (Figure 1B). Residual iris tissue and heavily pigmented trabecular meshwork fragments, which were identified as distinct black debris on the internal endothelial surface, were cleared by stabilizing the peripheral edge of the dome and gently agitating the tissue 3 to 5 times in sterile PBS. Any remaining adherent fragments were meticulously cleared using ultra-fine forceps tips (Figure 1C).

### 3.2. Profiles of Isolated Endothelial Sheets

Harvesting a clean and intact corneal dome is essential to preserve the intrinsic diversity, cellular sub-populations, and native spatial distributions of the corneal endothelial cell population. By initiating mechanical peeling from multiple discrete sites along the peripheral margins, the DM was gently and stepwise detached from the flapped limbal regions and advanced toward the central cornea. The entire endothelial sheet was successfully peeled off in a single continuous piece via slow, controlled movements using fine, non-toothed forceps (Figure 1D).

This mechanical, centripetal peeling technique successfully yielded intact endothelial monolayers from both age cohorts. Notably, the mechanical resistance was lower and the peeling motion was structurally smoother when utilizing tissues harvested from aged mice compared to young mice. The freshly isolated endothelium–DM complex naturally maintained an inward, dome-like physical curvature, which aided in distinguishing the anatomical orientations.

When the complex against the illumination bulb of a stereomicroscope, the cellular endothelial surface exhibited a characteristically dim, matte, and slightly irregular light reflection. In contrast, the acellular DM surface displayed a uniform, highly specular, mirror-like reflection (Figure 1E). Floating the isolated endothelium–DM complex in PBS under a light source effectively facilitated identification of the endothelial side, thereby ensuring that the acellular DM surface was positioned facing down toward the glass slide during subsequent whole-mounting.

### 3.3. Whole-Mounted Endothelial Sheet for Young and Old Mouse

Prior investigations predominantly utilized intact whole-mount corneal preparations to evaluate spatial and cellular features; however, the presence of the remaining epithelium and stroma significantly increased background noise signals and necessitated high-resolution imaging modalities. In contrast, the protocol optimized herein isolates a structurally isolated tissue layer, effectively resolving the phenotypic features of the corneal endothelium exclusively on the DM.

Following slow stepwise isolation, the primary corneal endothelium was whole-mounted onto glass slides using either fine-tipped forceps or a fine camel-hair painting brush. The fine brush worked more effectively to spread the elastic sheet evenly; however, inappropriate handling, such as contacting the tissue with the brush body rather than the ultra-fine tip, frequently resulted in severe localized cell loss at the peripheral zones where physical contact occurred (Figure S1).

**Figure 2:**
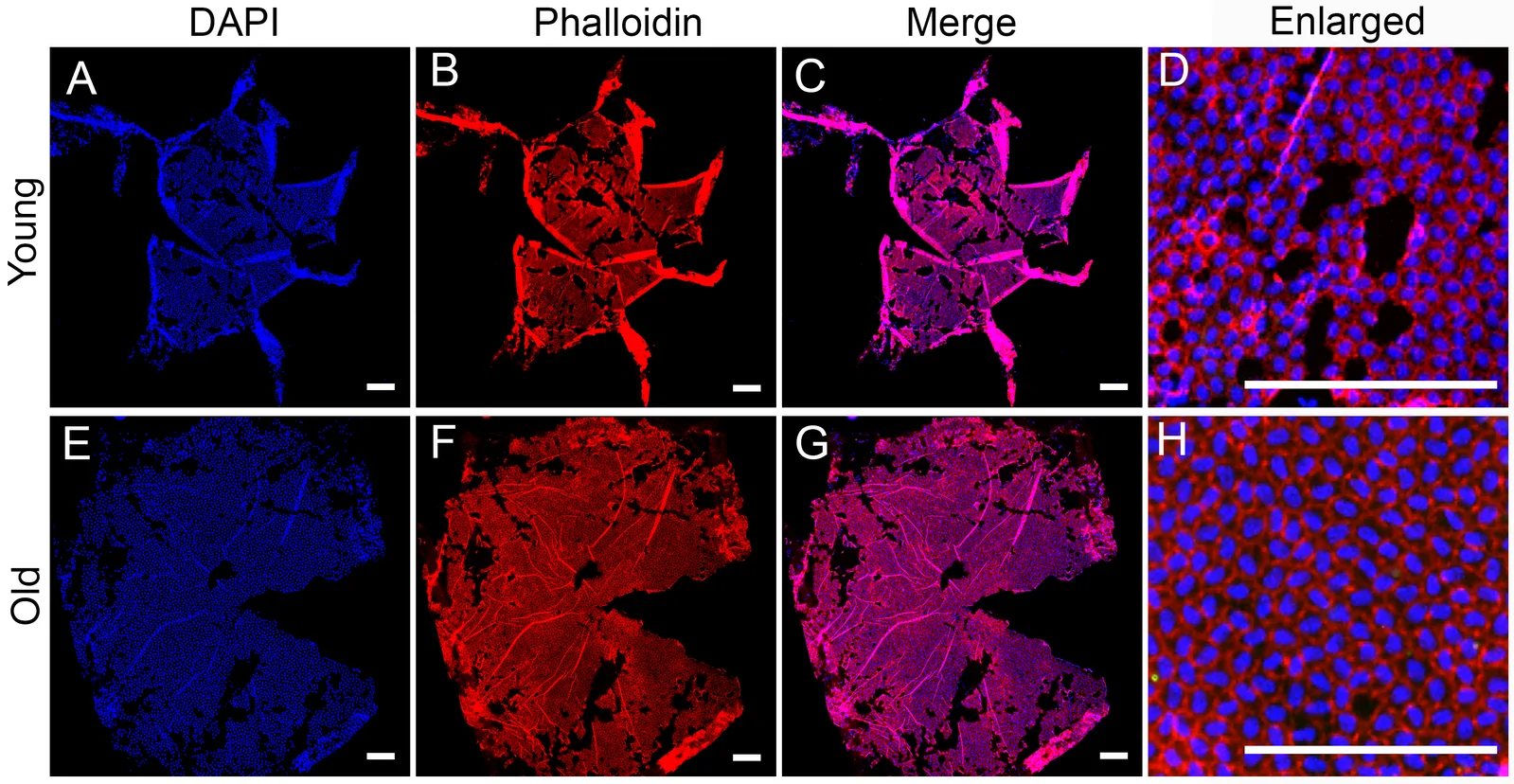
The whole-mounted corneal endothelium on Descemet’s membrane from young (**A**–**D**) and old (**E**–**H**) mice. Peeled endothelium was unfolded gently on a microscope slide under a dissection microscope, fixed with 4% paraformaldehyde, and stained with phalloidin-AF555 (**B**,**F**) and DAPI (**A**,**E**). (**C**,**G**) The merged images of DAPI and phalloidin-AF555. (**D**,**H**) A higher magnification of endothelium from （**C**,**G**), respectively. Scale bar = 200 μm.

The whole-mounted slide preparations permitted direct comparative analyses of cell size, cell density, and protein expression levels via immunofluorescence imaging. When comparing the isolated endothelium between young and aged mice, the corneal endothelial sheets were successfully flattened into a characteristic “flower-shaped” geometry on the slides (Figure 2A,E), effectively preserving the native polygonal mosaic morphology as visualized by Phalloidin-labeled F-actin mapping (Figure 2B,F). The vast majority of endothelial cells remained securely adhered to the DM substrate (Figure 2D,E), which indicated that cell loss was minimized during the mechanical peeling and flattening phases. Minor focal areas of cell depletion were restricted to the outermost limbal margins, which was attributed to direct mechanical trauma from the forceps and brush tips during tissue manipulation (Figure 2C,G). Nevertheless, cell–cell junctional integrity was structurally well-preserved and easily resolved in both young and aged murine cohorts (Figure 2D,H).

### 3.4. Ex Vivo Tissue Culture of Isolated Endothelial Sheet

**Figure 3:**
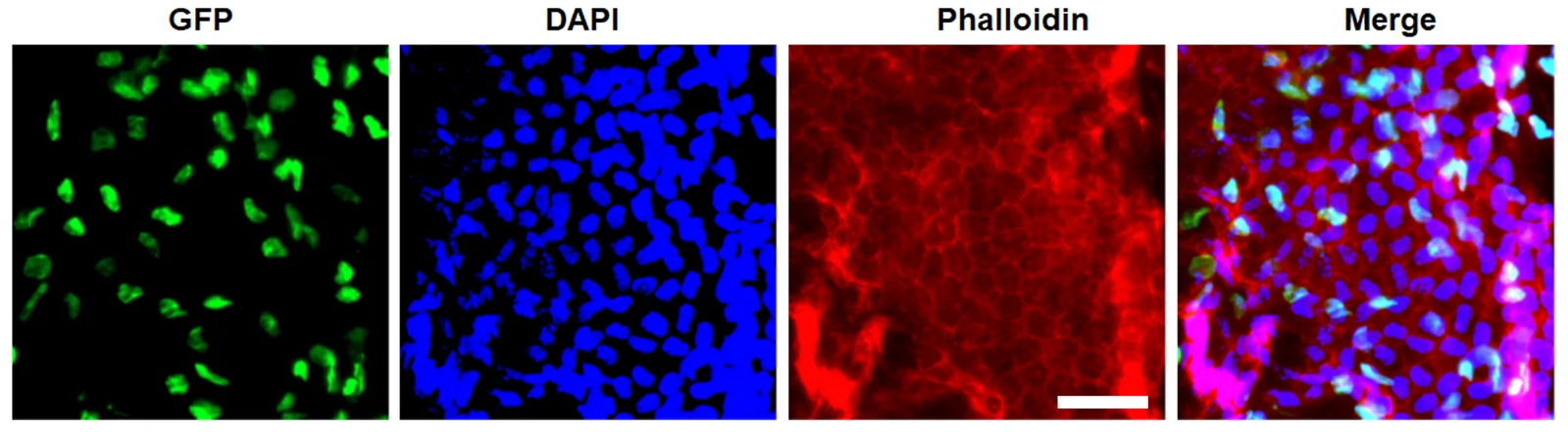
*Ex vitro* culture of isolated endothelium sheet. The isolated endothelial sheet was incubated overnight with 2 ug/mL doxycycline to induce the Tet-on-driven GFP expression. Cellular architecture and nuclei were resolved following a 5 min incubation with Phalloidin and DAPI prior to washing and whole-mounting. The sheet was then whole-mounted and imaged. Scale bar = 50 μm.

The isolated sheets demonstrated robust structural resilience, maintaining an intact cellular morphology over extended ex vivo handling. The endothelium–DM sheet could be preserved in its native dome shape during organ culture incubation for at least 24 h, and reporter transgene expression was successfully activated within hours of incubation (Figure 3). Following incubation, cell morphology and cellular junctions remained clearly defined, as verified by Hoechst 33342 or Phalloidin staining.

Compared to freshly isolated endothelial sheets, overnight organ culture incubation in medium containing 20% FBS did not induce any statistically significant reduction in cell viability (90.4 ± 2.8 vs. 94.5 ± 1.8%, *p* = 0.35, paired *t* test, as shown in Figure S2).

Furthermore, following enzymatic removal of the corneal epithelium, the peeling of the corneal endothelium enabled the parallel isolation of the underlying stromal dome. This isolated corneal stroma was successfully maintained in tissue culture for downstream applications, including the induction of doxycycline-inducible (*Tet-On*) gene expression patterns (Figure S3).

### 3.5. Isolation of Primary Endothelial Cells in Single-Cell Suspension

**Figure 4:**
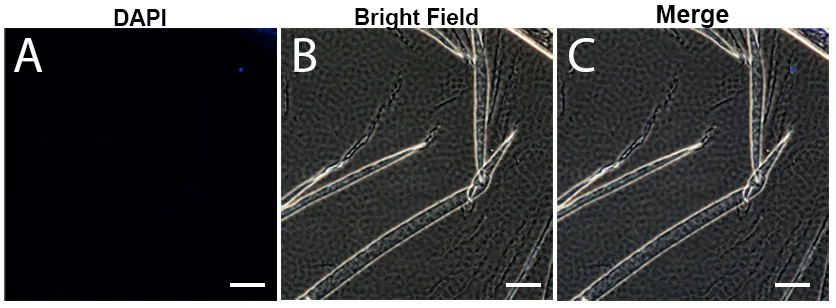
Valuation of the remaining corneal Descemet’s membrane (DM) following primary cell isolation. (**A**) Fluorescence microscopy showing nuclear counterstaining with DAPI, confirming complete cellular clearance with only a single remaining nucleus retained on the isolated basement membrane by trypsinization. (**B**) Brightfield micrograph resolving the structural layout of the remaining DM sheet. (**C**) Merged overlay of the DAPI and brightfield channels. Bar scale = 100 μm.

Single-cell RNA sequencing (scRNA-seq) technologies are increasingly utilized to map distinct cellular profiles, functional states, and transcriptomic signatures in both unicellular and multicellular tissues. And we were able to identify novel progenitors from limbal stem cells by scRNA-seq [45]. In this study, an adapted single-cell isolation workflow was established to evaluate the corneal endothelium at a single-cell level, further enabling the heterogenous profiling of corneal endothelium. The peeled endothelial sheet, along with its underlying DM, was enzymatically dissociated into a single-cell suspension using 0.25% Trypsin-EDTA. Following an 8 min incubation at 37 °C, the primary endothelial cells were detached from the DM via gentle mechanical pipetting. Complete cellular detachment from the extracellular matrix was confirmed by counterstaining the remaining matrix sheets with DAPI, which revealed a total absence of nuclei (Figure 4).

**Figure 5:**
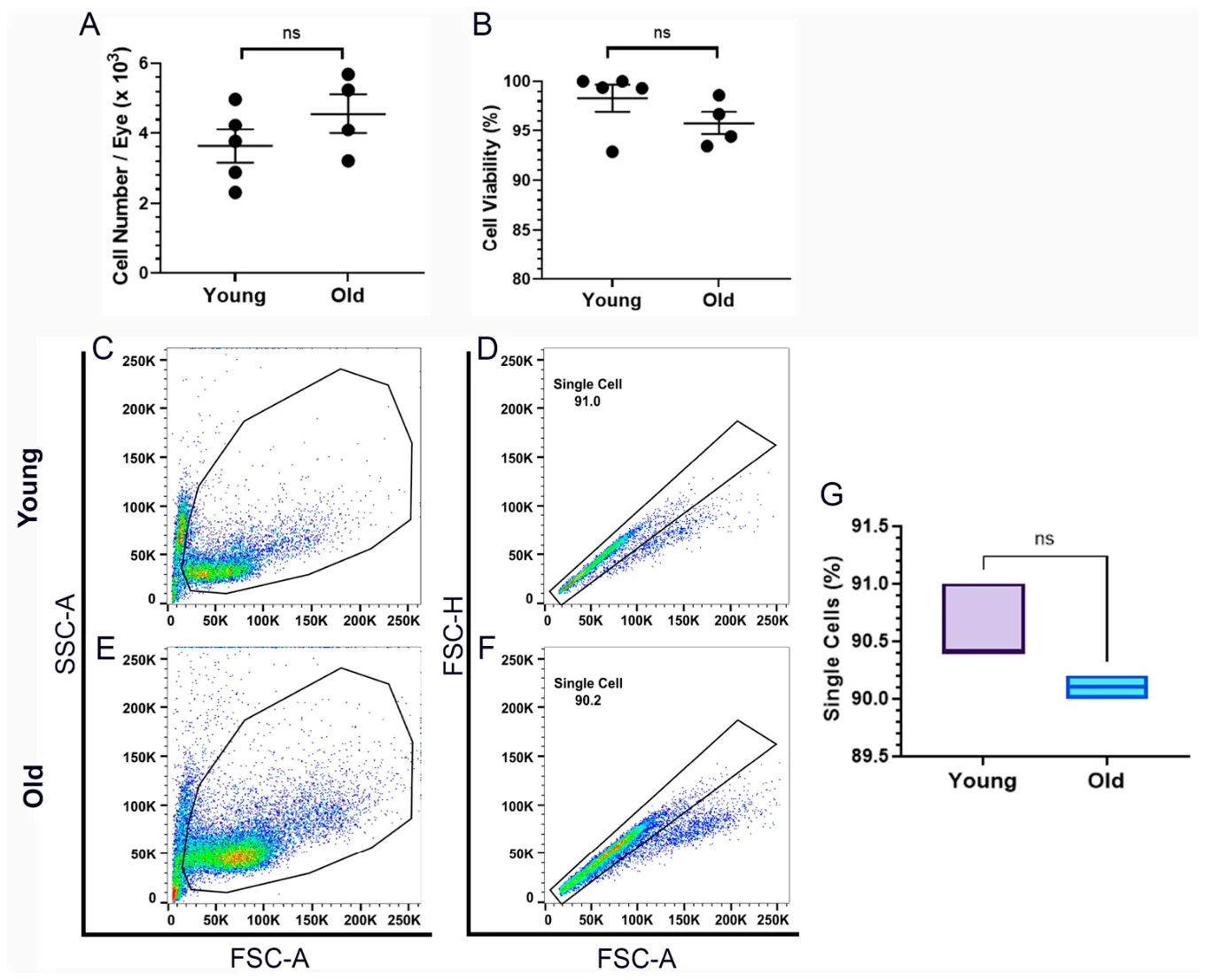
Quantitative evaluation of cell yield, viability, and singlet efficiency of primary corneal endothelial cells isolated from young (n = 5) and aged (n = 4) mice. (**A**) The total number of primary corneal endothelial cells collected per eye from young and old mice. (**B**) Cell viability profiling demonstrates the percentage of live corneal endothelial cells collected from young and old mice. (**C**–**G**) Flow cytometry assessment of single-cell suspension efficiency. Forward scatter versus side scatter (FSC vs. SSC) density dot plots derived from the single-cell suspensions of young (**C**) and aged (**E**) mouse corneas. Singlet gating profiles showing the precise single-cell populations isolated within the rectangular gating fields for young (**D**) and aged (**F**) samples. Statistical evaluation of singlet efficiency, confirming a high percentage of true single cells recovered from both experimental cohorts. Bars represent mean ± SEM. ns indicates *p* > 0.05.

This enzymatic dissociation protocol performed reliably for both young (1-month-old) and aged (1-year-old) mouse corneas. The average cell yields collected per eye were 3634 cells for young mice and 4560 cells for aged mice (Figure 5A), with high post-isolation cell viabilities of 98.3% maintained in the young cohort and 95.8% in the aged cohort (Figure 5B). Quantitative single-cell efficiency profiles were derived from flow cytometry data for both age groups (Figure 5C–F). The percentage of singlets obtained from young mice was 90.6%, and that from aged mice was 90.1% (Figure 5G).

**Figure 6:**
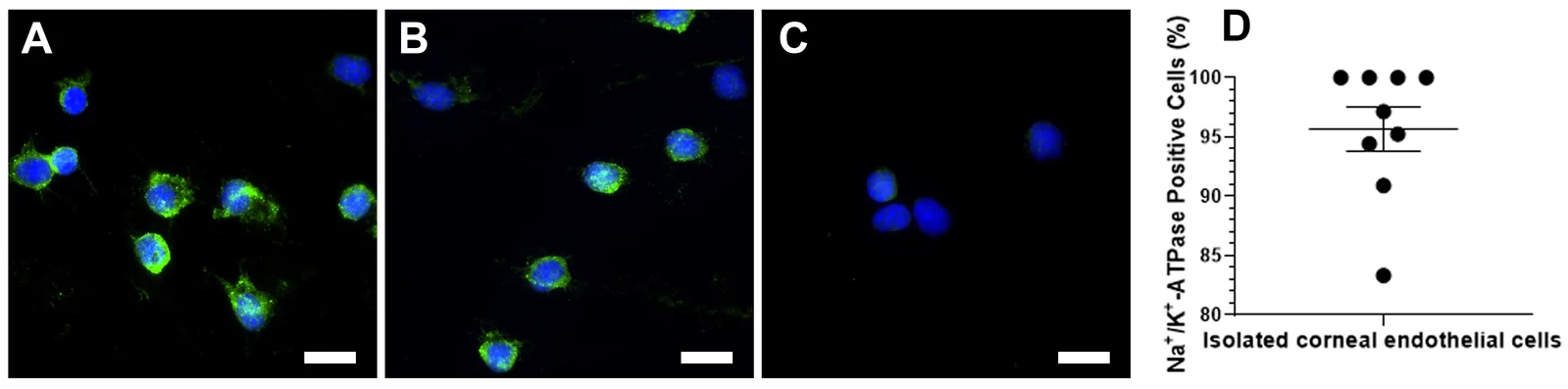
Immunocytochemical validation of phenotypic cell purity. (**A**,**B**) Fluorescence micrographs of isolated primary cells evaluated for the expression of the definitive functional marker Na^+^/K^+^-ATPase (green) and counterstained with DAPI (blue). (**C**) Negative control of the immunocytochemistry with the omission of the primary antibody. (**D**) Quantitative analysis of the percentage of Na^+^/K^+^-ATPase positive cells in the isolated corneal endothelial cells. Error bars represent SEM. N = 9; Scale bar = 20 μm.

**Figure 7:**
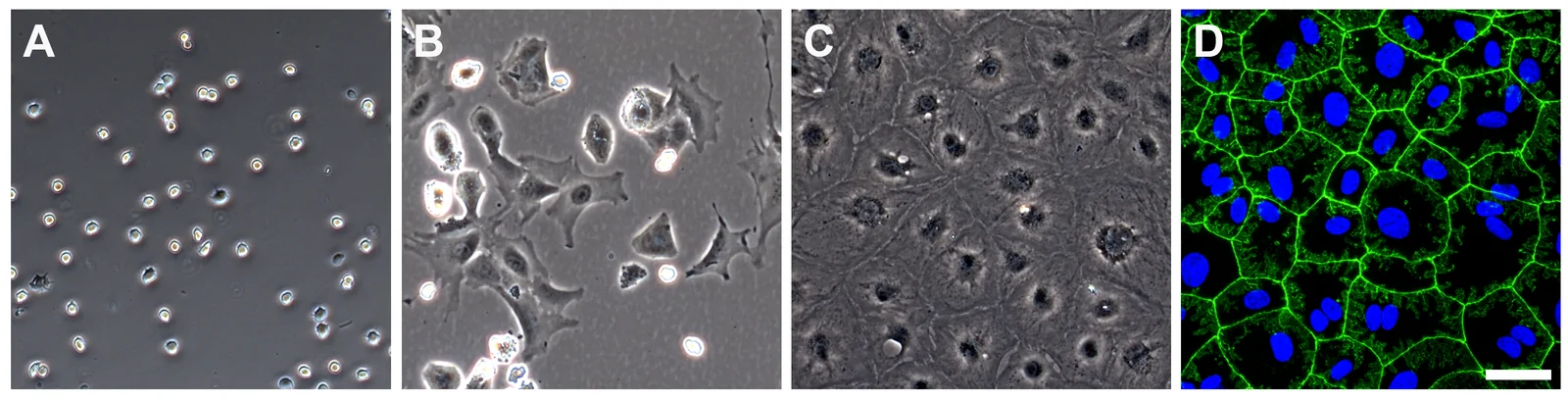
Primary culture of isolated mouse corneal endothelial cells. (**A**) Freshly isolated corneal endothelial cells from a young wild-type mouse (5 months old) seeded on a tissue culture chamber slide. Representative images of cultured endothelial cells on a glass chamber slide at (**B**) day 3 and (**C**) day 12 post-seeding. (**D**) Immunofluorescence staining of N-cadherin (green) counterstained with DAPI (blue) to visualize nuclei on day 20. Scale bar = 50 μm.

To further analyze the cell purity of isolated corneal endothelial cells, the cells were examined for the expression of the definitive functional marker Na^+^/K^+^-ATPase by immunocytochemistry (Figure 6). Approximately 95% of the collected cells were strongly expressing Na^+^/K^+^-ATPase localized to the plasma membrane (Figure 6A,B), indicating a high phenotypic purity of isolated corneal endothelial cells with negligible contamination from adjacent cell layers. Additionally, the isolated singlets were successfully re-cultured in glass chambers for downstream functional assays, retaining robust membrane localization of N-Cadherin (Figure 7).

This optimized isolation procedure provides a highly reliable primary cell resource to investigate the cellular heterogeneity of the corneal endothelium and advance the current understanding of endothelial development and cellular senescence during aging.

## 4. Discussion

Pre-clinical evaluation of novel therapeutic modalities for corneal diseases relies heavily on representative, well-characterized small animal models [46]. Although mechanical peeling techniques to harvest corneal endothelial sheets have been thoroughly optimized for large-eye models, such as human [44,47,48], non-human primate [49], and bovine tissues, applying these techniques to murine eyes has historically been limited by severe technical challenges. This difficulty is largely driven by the distinct micro-scale dimensions of the mouse ocular anterior segment; specifically, the mouse possesses a highly fragile ocular globe and an exceptionally thin Descemet’s membrane [50,51]. While the average thickness of Descemet’s membrane is 10.1 μm in humans [52,53]; however, the total DM thickness is about 1.83 μm in 6-week-old C57BL/6J mice and only 3.38 μm in aged cohorts [50]. Thus, this extreme thinness drastically increases the risk of tearing the extracellular matrix sheet during mechanical manipulation, leading to fragmented, low-yield tissue collections [15,27,37,54].

The protocol optimized in this study successfully overcomes these physical limitations, yielding highly enriched, viable, and structurally sound primary murine corneal endothelial sheets. Interestingly, a clear age-dependent variation was observed during micro-surgical execution: the peeling process was notably smoother and met less mechanical resistance in aged tissues (12 months old) than in young tissues (1 month old). This observation aligns with the documented age-related thickening of the basement membrane, which enhances the tensile strength of the DM–endothelial complex and reduces its susceptibility to focal tearing under directional mechanical force. Moreover, the quantitative data revealed that while aged eyes yielded slightly higher total cell count per cornea, the cell viability was slightly lower in the aged cohort. This marginal decline in viability is likely explained by an increased vulnerability of aged endothelial cells to physical stress during ex vivo manipulation, a phenomenon that remains consistent with previous reports on donor age-related cellular fragility [55,56].

Achieving high phenotypic purity requires the total removal of adhering uveal tissues, specifically the iris and trabecular meshwork (TM) fragments. Complete removal of these tissues was achieved by stabilizing the peripheral margin of the corneal dome with fine forceps while utilizing a second pair of ultra-fine forceps to grasp and strip the unwanted iris and TM remnants off the limbal rim through a firm one-time peeling. Another major source of cellular contamination originates from the corneal stroma, particularly within the peripheral areas where the peeling plane is initiated. If the initial micro-surgical incision plane extends too deeply into the limbal stroma, stromal keratocytes are inadvertently co-peeled, which can be identified visually by a distinct loss of transparency in the isolated sheet.

Our protocol addresses this challenge by utilizing a localized, multi-site approach. If stromal engagement is detected or suspected based on increased mechanical resistance or a localized decrease in tissue transparency, the operator immediately halts peeling at that position and re-initiates the plane of separation from a different clock position along the limbal rim. Minor stromal remnants can be carefully removed via gentle scraping with a fine forceps tip. This method significantly minimizes the cellular contamination that otherwise compromises downstream molecular profiles. Alternatively, the corneal dome can be incubated overnight to visualize cellular architecture via nuclear staining with 4 ng/mL Hoechst 33342, allowing for precise downstream isolation. Furthermore, this method can be extended to sequential stroma dome isolation and transgene activation; following the mechanical removal of both the epithelium and endothelium, the remaining corneal stroma dome demonstrates robust activation of reporter transgene expression (Figure 3 and Figure S3) upon incubation with 2 μg/mL doxycycline (lower than the oral delivery route in mouse).

Recent high-resolution single-cell surveys have revealed unexpected transcriptomic heterogeneity and distinct sub-population signatures within the human corneal endothelium [57], which was traditionally considered a homogeneous cell monolayer. Dissociation of the corneal endothelial sheet into a single cell suspension enables the comprehensive analysis of endothelial cells at the single-cell level. However, capturing high-quality single cells is essential yet technically challenging for scRNA-seq, requiring stringent protocols to enrich the target population while maintaining excellent viability [58,59]. Crucially, cell viability, the percentage of true singlets, and overall phenotypic purity are the primary determinants of downstream data accuracy. In the present protocol, utilizing the cleanly peeled endothelium–DM sheet allowed for the harvest of a highly purified population expressing membrane-localized Na+/K+ ATPase in more than 95% of the cells. On average, high single-cell yields (3634 to 4560 cells/eye) were successfully collected across both young and old mouse cohorts while maintaining elevated post-isolation viabilities (95.8% to 98.3%) and an excellent singlet efficiency exceeding 90%. This optimized yield and quality are highly beneficial for mapping distinct cell clusters and studying age-related endothelial decline in murine models. Furthermore, this methodology provides an efficient primary cell resource for evaluating advanced regenerative therapeutics, such as bioengineered cell-injection therapies.

Despite its high reproducibility, several limitations of this protocol must be acknowledged. First, the surgical procedure exhibits a high degree of operator dependency. It requires precise manual practice to master the limbal micro-scratching and centripetal peeling steps without puncturing or tearing the ultra-thin membrane. Second, although stromal contamination is actively managed by modifying the peeling sites, minor contamination from peripheral keratocytes may still persist at the molecular detection limit. Third, because of the small surface area of a single murine cornea, the absolute cell yield per eye remains low (3600 to 4600 cells), which demands careful pooling strategies when setting up downstream molecular or biochemical assays in younger cohorts.

## 5. Conclusions

In summary, this study provides an optimized, highly reproducible micro-surgical protocol for isolating primary mouse endothelial cells from both young and aged cohorts. By establishing a systematic method for limbal micro-scratching and centripetal mechanical peeling, this protocol successfully addresses historical challenges associated with low cellular yield, thin membrane fragmentation, and non-endothelial tissue contamination. The isolated endothelial sheets can be flattened successfully to preserve spatial cell-to-cell junctions and native polygonal mosaic morphology, or they can be dissociated into highly viable single-cell suspensions. Ultimately, this methodology offers a robust technical foundation for future translational ophthalmic research, enabling investigators to leverage single-cell transcriptomic and spatial profiling approaches to study corneal endothelial diseases, senescence, and regenerative medicine pipelines.

## Data Availability

The data presented in this study are available on request from the corresponding author.

## Supplementary Materials

The following supporting information can be downloaded at: https://www.mdpi.com/article/10.3390/bioengineering13080869/s1. Figure S1. Representative pictures showing a severe loss of endothelial cells at peripheral areas where the brush touched. (A) A whole-mount picture of corneal endothelial sheet labeled with DAPI. Scale bar = 200 μm. (B) An enlarged picture of the red box in A. Scale bar = 50 μm; Figure S2. Cell viability comparation before and after overnight incubation. The endothelial sheet peeled from corneal dome were directly mounted to slides with a drop of 0.4% Trypan blue for cell viability count. 4 random areas were selected, and cells were counted as blue-stained cells were thought to be dead cells. Paired t.Test was used for the comparison. *p* = 0.347; Figure S3. Tetracycline-inducible (Tet-On) GFP expression in the isolated corneal stromal disc during ex vivo organ culture. Following enzymatic digestion and removal of the corneal epithelium using Dispase I and Collagenase A, the corneal dome was excised from the ocular globe, and the endothelium-DM complex was removed via centripetal mechanical peeling. The remaining stroma dome was trimmed and incubation overnight in medium supplemented with 2 ug/mL doxycycline (Dox) to activate the GFP expression. Magnification: 4x; Table S1. Materials list.
