# Supplementary material for: An Optimized Mechanical Peeling Protocol for Mouse Corneal Endothelium
Source: Bioengineering (Basel). 2026 Jul 28;13(8):869. doi: 10.3390/bioengineering13080869 (PMC13510045; doi:10.3390/bioengineering13080869)
Supplement: Supplementary file 1 [file bioengineering-13-00869-s001.zip › bioengineering-4401477-supplementary.pdf]

Supplemental figures

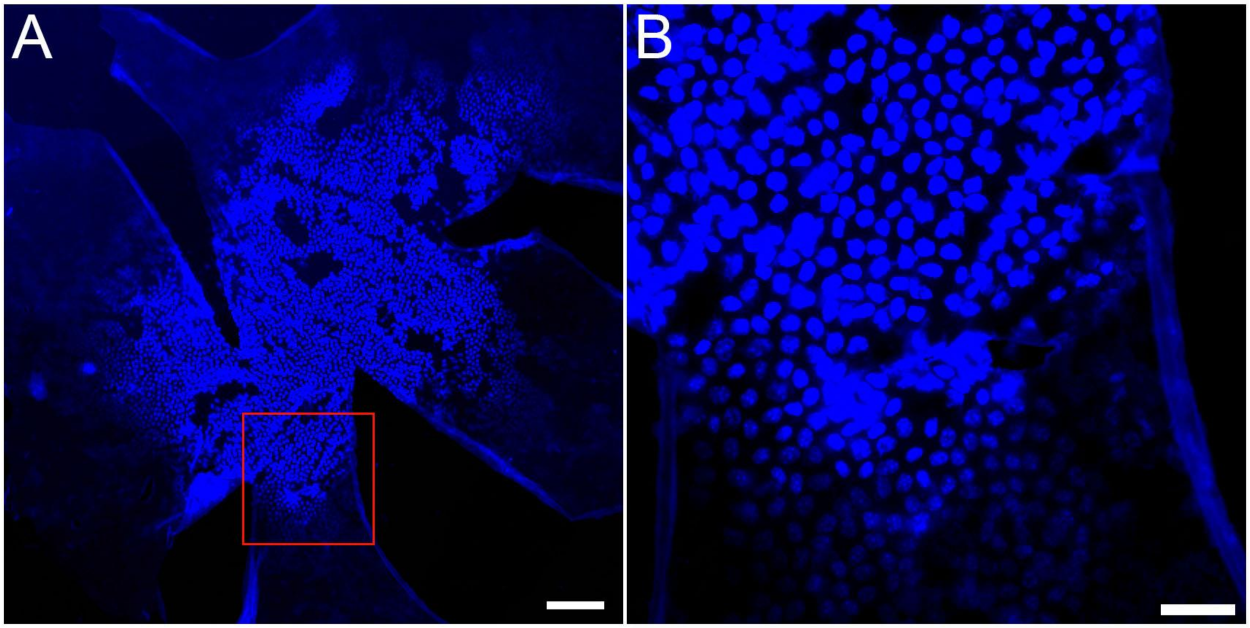

Figure S1. Representative pictures showing a severe loss of endothelial cells at peripheral areas where the brush touched. (A) A whole-mount picture of corneal endothelial sheet labeled with DAPI. Scale bar=200μm. (B) An enlarged picture of the red box in A. Scale bar=50μm.

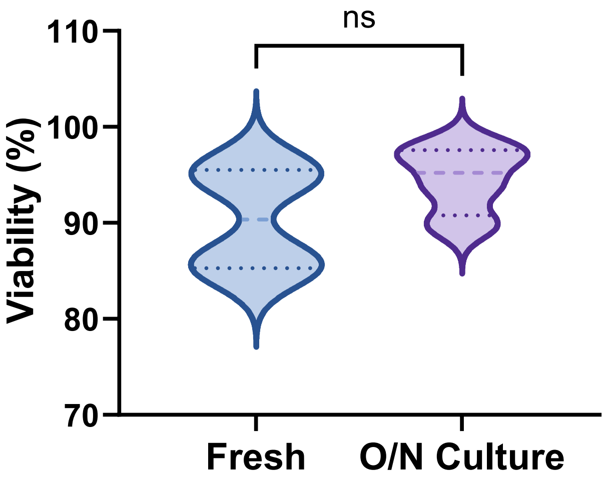

Figure S2. Cell viability comparation before and after overnight incubation. The endothelial sheet peeled from corneal dome were directly mounted to slides with a drop of 0.4% Trypan blue for cell viability count. 4 random areas were selected, and cells were counted as blue-stained cells were thought to be dead cells. Paired t.Test was used for the comparison. *P*=0.347.

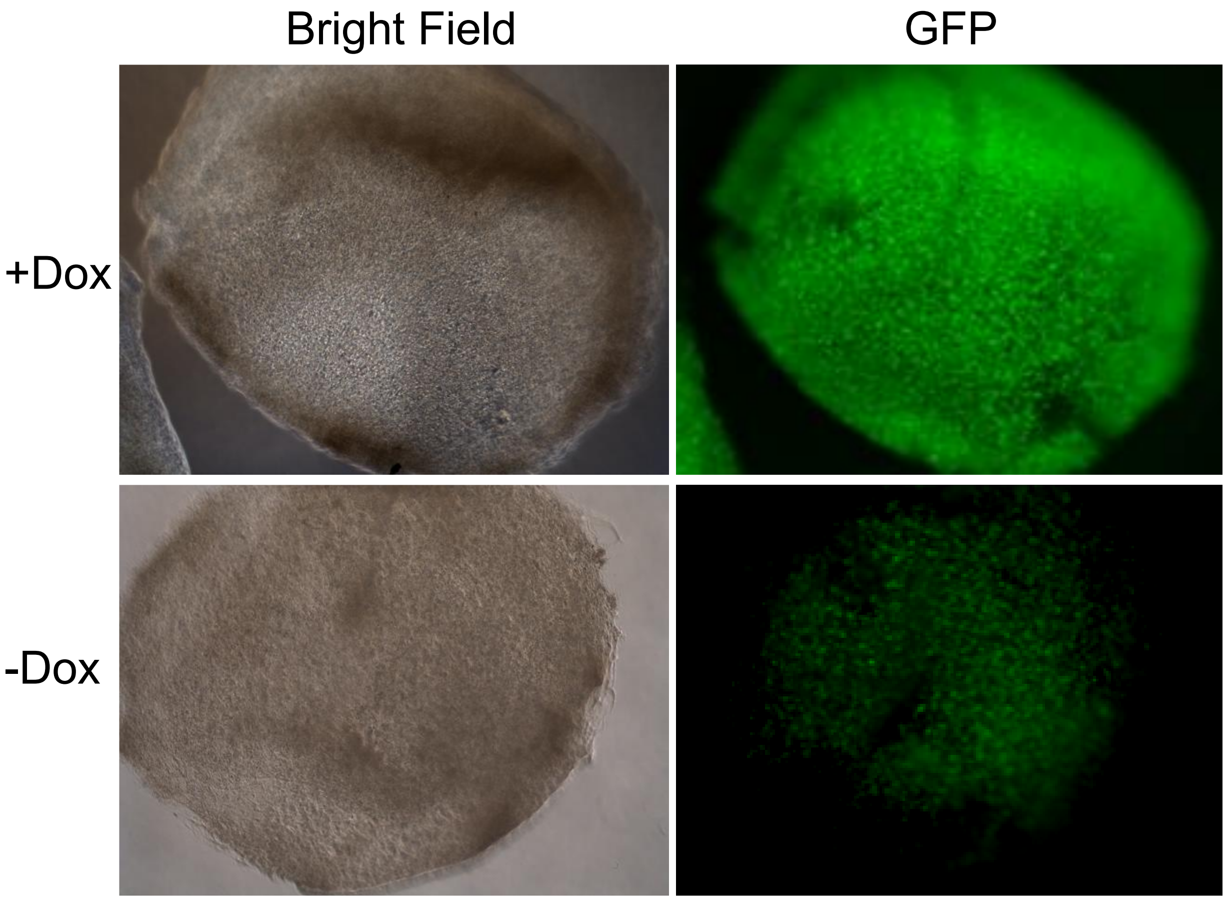

Figure S3. Tetracycline-inducible (Tet-On) GFP expression in the isolated corneal stromal disc during ex vivo organ culture. Following enzymatic digestion and removal of the corneal epithelium using Dispase I and Collagenase A, the corneal dome was excised from the ocular globe, and the endothelium-DM complex was removed via centripetal mechanical peeling. The remaining stroma dome was trimmed and incubation overnight in medium supplemented with 2ug/mL doxycycline (Dox) to activate the GFP expression. Magnification: 4x.

Table S1 Materials list

**Name of Material/Equipment**	**Company**	**Catalog Number**
C57BL/6J	Jackson Laboratory	000664
TetOP-H2B-GFP	Jackson Laboratory	016836
DMEM/F12 medium	Gibco	11330032
penicillin and streptomycin	Gibco	15-140-122
gentamicin/ amphotericin	Gibco	NC0308445
stereoscope	Leica	Mz6

Camera	Canon	T5i
Razor Blades	Fisher	12460
35mm Petri dish	Corning	353001
Straight fine forceps	RWD	F11020-11
Curved forceps	sklar	82030-028
Spring scissor, stright	RWD	S11020-11
Spencer Stitch Scissors, curved	Acuderm	A1301-090
Poly-lysine coated Slides	Fisher	1254578
Painting brush, 20/0	Artist's Loft	10213645
Kim paper	Kimberly-Clark	34155
fetal bovine serum, FBS	Sigma	TMS-013-B
phosphate buffered saline (PBS) buffer	Sigma	D8662
goat serum	Sigma	G6767
10% triton X100	Sigma	93443
Antifade Mountant with DAPI	Invitrogen	P36962
0.25% Trypsin/EDTA	Gibco	25200056
0.4% Trypan blue Solution	Gibco	15250061
16% Paraformaldehyde	Electron Microscopy Sciences	15710S
N-Cadherin (Rabbit mAb)	Cell Signaling Technology	13116s
Phalloidin-Alexa Fluor 555	Cell Signaling Technology	8953s
antibody Na+/K+ ATPase alpha1	Santa Cruz	sc-21712
Goat anti-Mouse IgG (H+L), AF488	Invitrogen	A-11029
Goat anti Rabbit IgG H&L, Alexa Fluor 488	Abcam	ab150117
Propidium Iodide, PI	Invitrogen	R37169
